# Usability Testing of a National Substance Use Screening Tool Embedded in Electronic Health Records

**DOI:** 10.2196/humanfactors.5820

**Published:** 2016-07-08

**Authors:** Anne Press, Catherine DeStio, Lauren McCullagh, Sandeep Kapoor, Jeanne Morley, SBIRT NY-II Team, Joseph Conigliaro

**Affiliations:** ^1^ Hofstra Northwell School of Medicine Manhasset, NY United States

**Keywords:** clinical decision support, adoption, primary care, usability, SBIRT

## Abstract

**Background:**

Screening, brief intervention, and referral to treatment (SBIRT) is currently being implemented into health systems nationally via paper and electronic methods.

**Objective:**

The purpose of this study was to evaluate the integration of an electronic SBIRT tool into an existing paper-based SBIRT clinical workflow in a patient-centered medical home.

**Methods:**

Usability testing was conducted in an academic ambulatory clinic. Two rounds of usability testing were done with medical office assistants (MOAs) using a paper and electronic version of the SBIRT tool, with two and four participants, respectively. Qualitative and quantitative data was analyzed to determine the impact of both tools on clinical workflow. A second round of usability testing was done with the revised electronic version and compared with the first version.

**Results:**

Personal workflow barriers cited in the first round of testing were that the electronic health record (EHR) tool was disruptive to patient’s visits. In Round 2 of testing, MOAs reported favoring the electronic version due to improved layout and the inclusion of an alert system embedded in the EHR. For example, using the system usability scale (SUS), MOAs reported a grade “1” for the statement, “I would like to use this system frequently” during the first round of testing but a “5” during the second round of analysis.

**Conclusions:**

The importance of testing usability of various mediums of tools used in health care screening is highlighted by the findings of this study. In the first round of testing, the electronic tool was reported as less user friendly, being difficult to navigate, and time consuming. Many issues faced in the first generation of the tool were improved in the second generation after usability was evaluated. This study demonstrates how usability testing of an electronic SBRIT tool can help to identify challenges that can impact clinical workflow. However, a limitation of this study was the small sample size of MOAs that participated. The results may have been biased to Northwell Health workers’ perceptions of the SBIRT tool and their specific clinical workflow.

## Introduction

### Screening, Brief Intervention, and Referral to Treatment: National Program that Works

Screening, brief intervention, and referral to treatment (SBIRT) is a nationally and federally sponsored program that provides a structured approach to better aid health care providers in identifying risky substance use and delivering early intervention and treatment services for persons at risk of, or with substance use disorders. SBIRT is an evidence-based protocol to identify patients who use substances in ways that increase their risk of health (physical or emotional), work, family, or social problems. It is used in a variety of settings including primary care practices, emergency departments, colleges, employee assistance programs, and mental health agencies. New York (NY)SBIRT-II is a project funded by the Substance Abuse and Mental Health Services Administration and coordinated by the NY State Office of Alcohol and Substance Abuse Services. Its goal is the implementation of a sustainable model for administering the SBIRT within New York State. Northwell Health and The National Center on Addiction and Substance Abuse at Columbia University are partners in the implementation of this project in three major Northwell Health departments; Emergency Medicine, Medicine, and Psychiatry/Behavioral Health.

With collaborative efforts between the Northwell Health Office of the Chief Information Officer and multiple teams within the Northwell Health Information Technology infrastructure, NYSBIRT-II has been successful in embedding SBIRT services within four major electronic health record (EHR) systems (AllScripts Electronic Health Record; AllScripts Emergency Department Information System; Sunrise Emergency Care). These EHR systems are used in both emergency medicine and primary care settings. These efforts have benefited the overall project by allowing the tool to integrate well within clinical workflows (which are heavily dependent on EHR usage).

### Electronic Screening, Brief Intervention, and Referral to Treatment: Success and Pitfalls

Since 2008, there has been an increase in the adoption of EHR technology to meet objectives set forth by the Health Information Technology (IT) for Economic and Clinical Health Act of 2009. The expansion of tools being developed in the EHR is allowing researchers and clinicians to find creative solutions for streamlining screening guidelines and standardizing care management plans [1].

While the SBIRT screen has traditionally been conducted through paper-based surveys administered by clinical staff, there is a growing emphasis on finding health IT strategies to integrate the screen into health technology platforms, such as the EHR.

### Formative Assessment and Usability Testing

There is an over arching goal among health systems to increase the use of the EHR in order to decrease medical errors and in turn, increase the quality of patient care. However, if the user is not taken into regard in the design of the tool, this may lead to failure of successful integration into the health care providers’ workflow. Ultimately, this can lead to EHR tools being neglected [2-4].

Researchers have now employed usability testing methods from the commercial industry and are applying them to health IT and EHR products with success [5-9]. Usability testing has shown to be successful in increasing a clinical decision support (CDS) tool’s use and impacting providers’ behavior. This is a result of formative assessment and usability testing addressing all components of the clinical environment and how the CDS tools address the micro (clinicians’ workflow) and macro (system) levels factors. These factors will impact the design and workflow of an EHR tool [10,11]. An example of factors to consider are the organizational policies around using pop-up alerts in the EHR (system), nurse’s versus clinician’s culture, and communication style during a clinical visit (clinical). On a personal level, each level of provider interacts with the EHR during a different decision making process and having the tool to execute during that specific point could determine acceptance or dismissal of the tool [12]. All of these challenges are addressed during the early stages of usability testing. Usability testing stresses iterative designs with the goal of creating tools that streamline care and improve compliance, while making the clinical visit more efficient [2].

Therefore, in designing a health IT solution for SBIRT, we sought out a formative assessment process and conducted several rounds of usability testing to determine the best design. We also sought to document the methods, strategies, and lesson learned from usability testing that could be shared nationally and guide others on their implementation strategy. This study evaluated the integration of an electronic SBIRT tool into clinical workflow. We hypothesized the electronic version would enhance the clinician workflow.

## Methods

### Study Design

**Figure 1:**
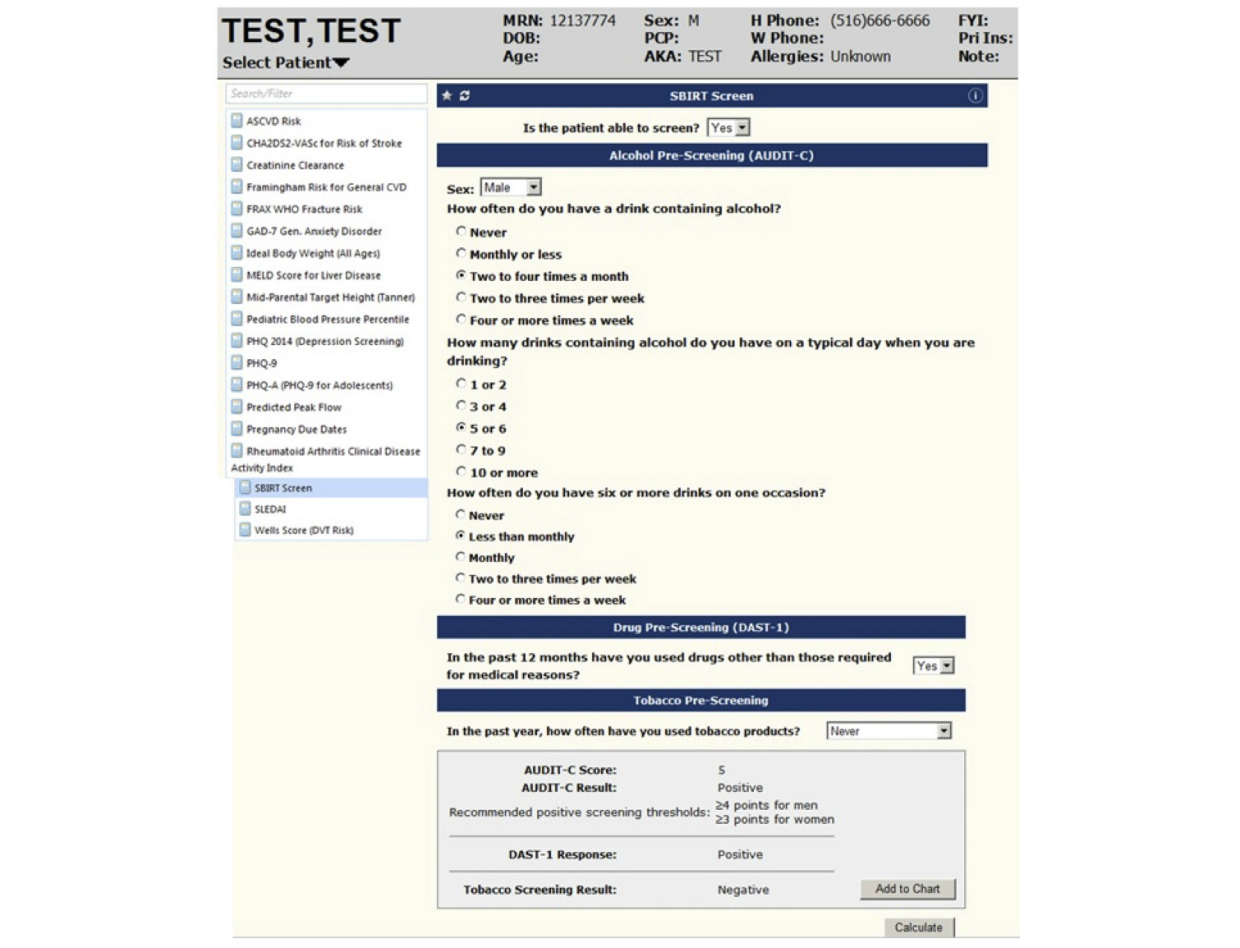
EHR SBIRT tool.

An observational study was conducted in an academic primary care practice (patient-centered medical home) within Northwell Health. The SBIRT screening tool had been originally implemented on paper and later implemented within the EHR system, which calculates the screening tool (Figure 1). The SBIRT tool is a screening tool that enables health coaches to identify those patients at risk for addiction in order to allow for early intervention and referral. Eligibility criteria for participation in this study included: employed as a medical office assistant (MOA) at the primary care clinic, past clinical experience, familiarity with the paper and electronic SBIRT screening tool, previous experience working with AllScripts EHR, and over the age of 18 years. A summary of participant demographics is summarized in Table 1.

**Table 1:** Medical office assistant demographics.

Participant	1 (Round 1)	2 (Round 1)	3 (Round 2)	4 (Round 2)	5 (Round 2)	6 (Round 2)	Average
Years worked as above title	2 years	14 years	5 years	2 years	3 years	3 years	4.8 years
Age	25 years	46 years	39 years	23 years	27 years	46 years	34.3 years
Years of medical experience	5 years	16 years	11 years	3 years	5 years	10 years	8.3 years
If yes to above, were you trained on computer and/or paper	Computer and paper	Computer and paper	Computer and paper	Computer and paper	Computer and paper	Computer and paper	6/6 (100%) computer and paper
Which SBIRT^a^ do you use regularly	Paper	Paper	Computer	Computer and paper	Computer	Computer and paper	2/6 (33%) paper 2/6 (33%) computer 2/6 (33%) computer and paper
How comfortable are you with SBIRT (1 to 5, with 5 people more comfortable)	5	5	5	5	5	5	5
Have you had experience in the past with computer decision support tools	No	Yes	No	No	Yes	Yes	3/6 (50%) yes 3/6 (50%) no
Have you had experience in the past with substance abuse screening tools	No	No	No	No	No	No	6/6 (100%) no
How long have you been using the above EMR^b^	2 years	4 years	4 years	2 years	3 years	3 years	3 years
How comfortable are you with the EMR you use? (1 to 5, 5 being most comfortable)	5	5	5	5	5	5	5

^a^screening, brief intervention, and referral to treatment.

^b^electronic medical record.

MOAs were given a description of this study and were given the opportunity to volunteer. All MOAs who volunteered had experience with SBIRT within Northwell Health. Two rounds of observations were conducted over a 1-year period. This research study has approval from the Northwell Health institutional review board.

### Description of the Tool

The SBIRT screening tool was built into the outpatient EHR system. In the first version, the tool was separated into two separate sections to be completed: alcohol and drugs was on one screen and tobacco was on a separate screen. In order to access the screens, you would have to click on each individually. Once each of the screens was completed, the user would have to make sure to click the “calculate” button. Once this was clicked, the user would then click the “add to chart” button. This had to be done on both the alcohol and drug screen and the tobacco screen. There was no alert system for the health coach once a screen had been added to the chart.

In the second generation of the tool, the questions were sectioned by category of alcohol, drugs, and tobacco; however, unlike the first version, they were all on the same page (Figure 1). The tool included the The Alcohol Use Disorders Identification Test (AUDIT-C) Drug Abuse Screening Test (DAST-1), and Alcohol Smoking and Substance Involvement Screening Test (ASSIST) screening questionnaires that screen for alcohol, drugs, and tobacco use, respectively (Multimedia Appendix 1). The tool incorporated branching logic, wherein if the answer to the first question of the AUDIT-C is “Never,” then the two remaining AUDIT-C questions will not come into view. Similarly, if the answer to the first question of the AUDIT-C is monthly or more, than the two remaining AUDIT-C questions will appear. Once calculate is pressed, the patient’s age, gender, and given answers are used to determine if the patient’s prescreen is negative or positive. Once the prescreen is complete it is saved under Order Viewer, CLINICAL Calculators, and summarizes the patient’s status (prescreen negative or positive), questions, and responses (Multimedia Appendix 2). If a screen were calculated as positive by the electronic SBIRT tool it would go into the user’s task list within the EHR. The user then forwarded the task to the health coach in order to alert the health coach of a positive screen.

### First Round of Testing

The first round of usability testing was conducted directly after an electronic-based screen was introduced into the EHR to frontline clinical staff who were using a paper-based workflow. During the first round, two participants volunteered for this study. The MOAs had received trainings on both the paper and electronic versions of the SBIRT screen and had approximately 1 week of familiarity with both mediums. Testing was done within the clinical setting in which the MOAs regularly work and a standardized patient was used for the clinical scenarios. One MOA interacted with a patient at a time and the patient presented the same case in both interactions. For the first interaction, the MOA completed the paper SBIRT screen and for the second interaction the MOA completed the electronic SBIRT screen. Each electronic screen consisted of two parts: part one for alcohol and drug use and part two for tobacco use. After completion of the interactions, the MOA submitted the screen depending on the existing workflow and the medium used – into the completion box for paper screens, and pressing the “add to chart” button for the electronic screening tool.

During the first round of testing, the MOAs were audio and video recorded and had a research staff member present for the interactions. Following the standardized patient visit, the MOAs participated in informant interviews regarding their experiences with the SBIRT screening tool, conducted by study staff. The interviews included qualitative questions and annotative feedback regarding the screening mediums.

**Figure 2:**
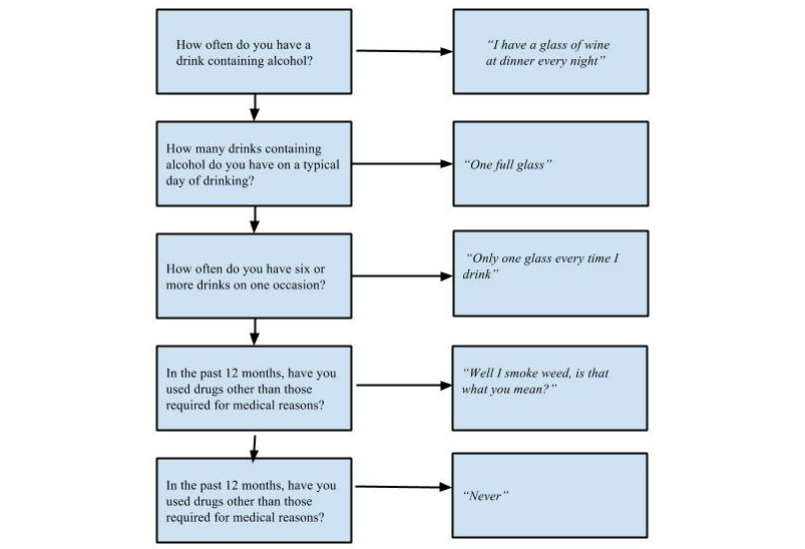
Mock patient scenario.

Recordings of the mock clinical sessions and annotations of the interviews were analyzed by two raters for thematic similarities. The analysis included both qualitative and quantitative measures based on the recordings of the clinical scenarios and the answers to the informant interviews. The quantitative measures analyzed were time and accuracy during the clinical scenarios, while the qualitative measures that were analyzed were the observations of the clinical scenario and MOA responses during the interviews. An example of patient interaction is seen in Figure 2.

### Second Round of Testing

The second round of testing took place 12 months later when version two of the electronic tool was integrated into the EHR. The second generation was created based on feedback from the first round of testing and MOA feedback. While awaiting the revision of the first generation of the electronic screen, paper screening continued to be used.

During the second round of testing, four MOAs volunteered to participate. The MOAs had 2 weeks of familiarity with version two of the electronic screen. The testing scenario was kept the same as it was in the first round. Important differences were as follows: (1) version two of the electronic screen was used, therefore there was only one part to the screen, as opposed to the two seen in the first round, and (2) there was no audio or visual recording in the second round. This was due to two research staff members being present for the interactions.

As in the first round of testing, following the standardized patient visit, the MOAs participated in informant interviews regarding their experiences with the SBIRT screening tool, conducted by study staff. The interviews included qualitative questions and annotative feedback regarding the screening mediums made by the MOAs. Annotations of the interviews were analyzed by two raters for thematic similarities. The analysis included qualitative measures based on the answers to the informant interviews. The qualitative measures that were analyzed were the observations of the clinical scenario and MOA responses during the interviews.

## Results

### First Round

Cases were analyzed using both quantitative and qualitative analyses. As part of the quantitative analysis, the two screen mediums were timed to compare efficiency of both SBIRT mediums. Timing was done using the time stamp on the recording device. The SBIRT screening took an average of 45 seconds when used as a paper screen and 94 seconds when the electronic version was employed. This results in a 48.5 second difference between the two mediums, with the paper screen being almost two times faster to complete than the electronic version.

The ability to complete the tool was evaluated as well. Each paper screen was completed correctly and submitted to the correct location (health coach completion box). The electronic screens were completed correctly, however, in both cases there was a failure to submit part one, the drug and alcohol section, to the correct location; clicking add to chart within the EHR was not done.

**Figure 3:**
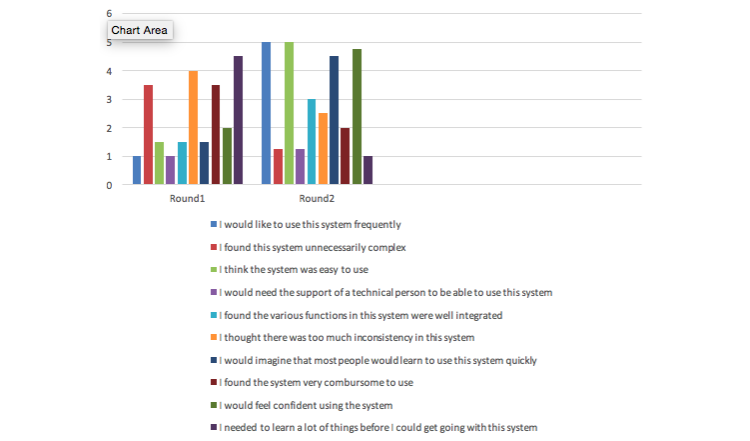
Comparison of the system usability scale between Round 1 versus Round 2 of the EMR SBIRT tool.

As additional quantitative analysis, the MOAs completed the system usability scale (SUS) survey [13] after the mock patient scenario was completed. Answers were totaled by percentages for each question (Figure 3). The results of the quantitative analysis are summarized in Table 2.

**Table 2:** Round 1: observations from qualitative analysis.

SBIRT^a^tool	Usability constructs (workflow integration, efficiency; effective; learnability; satisfaction)	Example rater comments
Paper Screen MOA^b^ #1	Good patient/provider interaction Strong workflow integration	Smooth flow between questions Eye contact A lot of patient interaction Very few interruptions with workflow
Paper Screen MOA #2	Good patient/provider interaction Strong workflow integration	A lot of patient eye contact, interaction, and engagement
Electronic screen MOA #1	Poor workflow integration Inefficient tool Poor patient/provider interaction	Slow transition between questions Slow to initialize EHR^c^tool after introducing the SBIRT tool to the patient Not a lot of eye contact Few direct patient interactions Struggled to find boxes to fill out the tool Difficulty calculating the score
Electronic screen MOA #2	Poor workflow integration Inefficient tool Poor patient/provider interaction	Not a lot of eye contact with the patient Not a lot of patient interactions Great difficultly flowing through the questions Needed guidance with regards to where to click

^a^screening, brief intervention, and referral to treatment.

^b^medical office assistant.

^c^electronic health record.

Areas for improvement of the SBIRT tool were suggested and included increasing spacing of the question and answers to avoid wrong clicking, one button to identify all answers as negative, keeping the order of questions the same as the paper version, faster loading process, and a change in the alerting system that ensures addition of the form to the chart.

After Round 1 of testing, conversations with the study team (clinical leaders, project directors, health coaches) and clinic staff deemed that the electronic screen tool was slower and less efficient that the paper version. This resulted in the immediate cessation of the EHR-based tool, and full paper-based workflow.

### Second Round

Cases were analyzed using both quantitative and qualitative analyses. As part of the quantitative analysis, MOAs completed the SUS survey after the mock patient scenario was completed. Answers were totaled by percentages for each question. Results show the highest rated answers were that the MOAs would use the system frequently, the system was easy to use, and the MOAs did not need to learn a lot to use the system. These questions all received a 100% rating. The lowest rated answers were that there is too much inconsistency within the system and all functions are not well integrated.

The ability to complete the tool was evaluated, as it was in the first round. All screens were completely correctly and submitted to the correct location (clicking add to chart within the EHR and alerting the health coach via EHR). The results of the qualitative questioning are summarized in Table 3.

**Table 3:** Qualitative data on usability constructs: paper versus electronic SBIRT tool.

Question	Round 1		Round 2	
	Paper version	Electronic version	Paper version	Electronic version
Did you make a lot of mistakes while you were working with the programs?	No	Yes	No	Yes-hit the wrong button especially when in a rush
How do the versions work in the daily workflow?	Convenient; easy to incorporate	Too much to navigate; scrolling and clicking takes a long time; gets in the way of normal workflow	More effective interaction with patient	Enter it into the EMR^a^ after patient seen; lag time in loading slows down efficiency; easier to not have the computer with you; difficult to do when there are multiple patients
What are some of the pros and cons/obstacles, etc that you have faced while working the tool?	More patient eye contact; easy and fast	Would prefer this version if it were easier to use; inconvenient; slow; interferes with patient interaction; less patient eye contact and interaction	Easy and fast; have hard copy in case something goes wrong with EMR; can talk to health coach about complex patients before entering; takes time to look up patient information to be added to sheet; have to remember to bring to health coach; waste of paper	Accuracy in EMR; easy to work with; goes right to patient chart and right to the health coach; everything automatically documented; easier to write on paper and transfer; slows down work flow; Interferes with patient interaction; lags when opening; order of questions is not the same as the paper was; can forget to click to calculate, add to the patient chart, or to alert the health coach.
Suggestions and improvements	If the electronic were easier to use, would prefer that; set up (in the EHR^b^) should be more like the paper; too many clicks with the electronic version		Improve layout/spacing of the question answers to avoid clicking wrong option; if patient is negative for everything – there should be one button to press so as not to waste time going through all questions; keep the order of the questions the same as when the original implementation was done (electronic version should have been the same as the paper version)	
Which method do you prefer?	2/2 (100%) paper		2/4 (50%) electronic 2/4 (50%) undecided	

^a^electronic medical record.

^b^electronic health record.

## Discussion

### Round 1

During Round 1 of usability testing, we observed more cons in the electronic version than originally hypothesized, with our hypothesis being that the electronic version would improve workflow and have high user satisfaction/usability. Participants’ negative feedback on the electronic SBIRT tool was: a lack of interaction between the MOA and the patient, a lack of certainty in the hand-off of the tool to the health coach, and a decrease in general usability of the screening tool. An additional problem noted during Round 1 was the time to complete the electronic screen and improper completion of the tool. From the screen capture analysis, there were many mistakes captured in the electronic screening tool. The MOAs had difficulty navigating through the EHR and often wrong buttons were pressed or the “submit to chart” button was not pressed, as it was not readily visible after completion. It was noted however, that if the items mentioned were adjusted, the electronic screen had the potential to impact the patient visit in a positive manner. One participant noted “having all the clinical information for each patient in one place would make for a more efficient patient visit.”

Based on feedback and analysis of results from usability testing, many changes were made to the electronic SBIRT screen within the EHR. After the first round of testing, the problem of uncertainty of the hand off to the health coach was alleviated by alerts being set up within the electronic SBIRT tool. Once the screen was completed, calculated, and added to the chart, a positive screen was automatically sent to the patient’s chart and the users task list. The user then forwarded an alert to the health coach.

### Round 2

As a result of the usability testing, identifying barriers to adoption in the tool, and addressing them in the new design, during Round 2 of testing (1 year later), the MOAs felt the electronic SBIRT tool had improved and had high levels of user satisfaction. Specifically, participants noted that the delivery of the screen to the health coach and the patient’s EHR was more accurate than even the paper version. However, during Round 2, the workflow with the EHR screening tool had changed. Instead of entering the information from the screening directly into the computer during a patient interaction, answers were added only after the MOA was finished in the room with the patient. As noted by the MOAs in the qualitative interview, although the functionality of electronic SBIRT tool had improved due to adjustments based on Round 1, the issue with the length of time loading the tool on the computer created a situation that deterred the MOA from entering the screen into electronic SBIRT tool in real time during the patient visit. This slowed down workflow considerably. Therefore, discussions after Round 2 were focused on improving lag times so that MOAs could enter information in real time in order to enhance workflow and decrease timing of patient visits.

This study demonstrates the importance of usability testing during initial design. We were able to refine an electronic SBIRT tool to address usability barriers and create a user-friendly version of the electronic tool. This study also demonstrates how clinical practice is dynamic, and therefore tools should also be flexible and easily edited. Usability testing a year later was able to identify new barriers and direct a new iteration of the tool. Using the SBIRT screening tool as a clinical case to constant usability testing highlights how other clinical decision support tools and electronic screening tools should consider periodic usability testing.

The SBIRT tool integration into the EHR also demonstrates that electronic tools may not always improve workflow if not tested thoroughly before implementation; electronic tools need a user-centered design before launching. The final electronic SBIRT tool sets precedence for other SBIRT sites to develop, test, and implement an electronic SBIRT tool in their EHR and clinical workflow.

### Limitations

One limitation of this study was the small sample size of MOAs that participated. The results may have been biased to Northwell Health workers’ perceptions of the SBIRT tool and their specific clinical workflow. Therefore, usability testing is recommended before the introduction of CDS and screening tools into each new environment, so tools can be customized to site specific and staff specific workflows.

### Conclusion

With the advances of health IT, progression of meaningful use, and implementation of EHR health systems, health systems are eager to implement IT solutions. The SBIRT screening tool is one example of this trend. Our study used a specific usability testing methodology to implement the SBIRT screening tool into the EHR in a way that was streamlined to the existing clinical workflow. Analysis of film, quantitative, and qualitative questions identified setbacks, areas for improvement, and highlights of both the first and second versions of the electronic SBIRT tool. By doing so we were able to identify ways to improve the tool, which resulted in an increase in user satisfaction and an increase in the accuracy of the tool. This study demonstrates the importance of usability testing in designing EHR tools. Future studies must focus on a more robust sample size in standardized usability testing laboratory to allow for more thorough investigations into the optimization of the SBIRT into its electronic form. An example of a study design would be talk aloud methodology combined with near-live simulation testing. This would allow for more individualized feedback and investigation into real-life workflow limitations and would allow for further optimization of the tool. This would also allow for revisiting fidelity, barriers/facilitators, and process mapping of clinical workflows at multiple time points.

## Appendix

Multimedia Appendix 1 AUDIT-C and DAST-1 questionnaire forms.

Multimedia Appendix 2 SBIRT screening tool workflow.

## Abbreviations

ASSIST: alcohol smoking and substance involvement screening test
AUDIT-C: the alcohol use disorders identification test
CDS: clinical decision support
DAST-1: drug abuse screening test
EHR: electronic health record
EMR: electronic medical record
IT: information technology
MOAs: medical office assistants
NYSBIRT: New York screening, brief intervention, and referral to treatment
SBIRT: screening, brief intervention, and referral to treatment
SUS: system usability scale
