# Supplementary material for: Usability Testing of a National Substance Use Screening Tool Embedded in Electronic Health Records
Source: JMIR Hum Factors. 2016 Jul 8;3(2):e18. doi: 10.2196/humanfactors.5820 (PMC4958139; doi:10.2196/humanfactors.5820)
Supplement: Multimedia Appendix 1 [file humanfactors_v3i2e18_app1.pdf]

Northwell Health

NEW YORK STATE Screening, Brief Intervention and Referral to Treatment

A Program of the New York State Office of Alcoholism and Substance Abuse Services

CASA COLUMBIA

Date:____________ **MRN______________________**

The Alcohol Use Disorders Identification Test: Interview Version

Read questions as written. Record answers carefully. Begin the AUDIT by saying, "Now I am going to ask you some questions about your use of alcoholic beverages during this past year." Explain what is meant by "alcoholic beverages" by using local examples of beer, wine, vodka, etc. Code answers in terms of "standard drinks". Place the correct answer number in the box at the right.

1. How often do you have a drink containing alcohol?:
   (0) Never [Skip to Qs 9-10]
   (1) Monthly or less
   (2) 2 to 4 times a month
   (3) 2 to 3 times a week
   (4) 4 or more times a week

2. How many drinks containing alcohol do you have on a typical day when you are drinking?
   (0) 1 or 2
   (1) 3 or 4
   (2) 5 or 6
   (3) 7, 8, or 9
   (4) 10 or more

3. How often do you have six or more drinks on one occasion?
   (0) Never
   (1) Less than monthly
   (2) Monthly
   (3) Weekly
   (4) Daily

4. How often during the last year have you found that you were not able to stop drinking once you had started?
   (0) Never
   (1) Less than monthly
   (2) Monthly
   (3) Weekly
   (4) Daily or almost daily

5. How often during the last year have you failed to do what was normally expected from you because of drinking?
   (0) Never
   (1) Less than monthly
   (2) Monthly
   (3) Weekly
   (4) Daily or almost daily

6. How often during the last year have you needed a first drink in the morning to get yourself going after a heavy drinking session?
   (0) Never
   (1) Less than monthly
   (2) Monthly
   (3) Weekly
   (4) Daily or almost daily

7. How often during the last year have you had a feeling of guilt or remorse after drinking?
   (0) Never
   (1) Less than monthly
   (2) Monthly
   (3) Weekly
   (4) Daily or almost daily

8. How often during the last year have you been unable to remember what happened the night before because you had been drinking?
   (0) Never
   (1) Less than monthly
   (2) Monthly
   (3) Weekly
   (4) Daily or almost daily

9. Have you or someone else been injured as a result of your drinking?
   (0) No
   (2) Yes, but not in the last year
   (4) Yes, during the last year

10. Has a relative or friend or a doctor or another health worker been concerned about your drinking or suggested you cut down?
   (0) No
   (2) Yes, but not in the last year
   (4) Yes, during the last year

	AUDIT	DAST
Neg	0-7	0
BI	8-15	1-2
EBI	16-19	3-5
RT	20+	6+

Record total of specific items here.

Date:____________ MRN________________________

DAST-10 Questionnaire

I'm going to read you a list of questions concerning information about your potential involvement with drugs, excluding alcohol and tobacco, during the past 12 months.

When the words "drug abuse" are used, they mean the use of prescribed or over-the-counter medications/drugs in excess of the directions and any non-medical use of drugs. The various classes of drugs may include: cannabis (e.g., marijuana, hash), solvents, tranquilizers (e.g., Valium), barbiturates, cocaine, stimulants (e.g., speed), hallucinogens (e.g., LSD) or narcotics (e.g., heroin). Remember that the questions do not include alcohol or tobacco.

If you have difficulty with a statement, then choose the response that is mostly right.
You may choose to answer or not answer any of the questions in this section.

**These questions refer to the past 12 months**		**No**	**Yes**
1	Have you used drugs other than those required for medical reasons?	0	1
2	Do you abuse more than one drug at a time?	0	1
3	Are you able to stop using drugs when you want to?<br>If never use drugs, answer "Yes". **(Note: reverse score for this question)**	**1**	**0**
4	Have you had "blackouts" or "flashbacks" as a result of drug use?	0	1
5	Do you ever feel bad or guilty about your drug use?<br>If never use drugs, choose "No".	0	1
6	Does your spouse (or parents) ever complain about your involvement with drugs?	0	1
7	Have you neglected your family because of your use of drugs?	0	1
8	Have you engaged in illegal activities in order to obtain drugs?	0	1
9	Have you ever experienced withdrawal symptoms (felt sick) when you stopped taking drugs?	0	1
10	Have you had medical problems as a result of your drug use (e.g., memory loss, hepatitis, convulsions, bleeding, etc.)?	0	1

**Score**	**Risk**	**Action**
0	Low Risk	Positive Reinforcement
1-2	At-Risk Use	Brief Intervention
3-5	Harmful Use	Referral to Brief Treatment
6+	Potential Dependent	Referral to Specialty Treatment

Total score

Northwell Health

NEW YORK STATE Screening, Brief Intervention and Referral to Treatment

A Program of the New York State Office of Alcoholism and Substance Abuse Services

CASA COLUMBIA

Date:____________ **MRN_______________________**

CUESTIONARIO DE USO DE DROGAS (DAST-10)

Las siguientes preguntas se refieren a información acerca de su potencial envolvimiento con drogas **excluyendo alcohol y tabaco** durante los últimos 12 meses. Lea con cuidado cada pregunta y decida si su respuesta es “No” o “Si”. Después, marque el casillero apropiado junto a la pregunta.

Cuando la palabra “abuso de drogas” es usada, esta se refiere al uso de medicinas compradas con receta medica o sin ella y que son ingeridas en exceso de lo recomendado. Las varias clases de drogas pueden incluir: cannabis (mariguana, hashis), solventes, tranquilizantes (Valium), barbitúricos, cocaína, estimulantes (speed), alucinógenos (LSD) o narcóticos (heroína). Recuerde que las preguntas **no incluyen** alcohol o tabaco.

Por favor responda cada pregunta. Si tiene dificultades con alguna de las preguntas, escoja la que le parezca correcta.

Marque “1” en la casilla correcta.

**Estas Preguntas Están Referidas a los Últimos Doce Meses:**	**No**	**Si**
1. ¿Ha usado drogas que no eran requeridas por razones medicas?	0	1
2. ¿Ud. abusa mas de una droga a la vez?	0	1
3. ¿Es Ud. capaz de parar de usar drogas siempre cuando se lo propone?**(reverse score)**	**1**	**0**
4. ¿Ha tenido "perdidas de conocimiento" o una "memoria repentina” como resultado del uso de drogas?	0	1
5. ¿Alguna vez se siente mal o culpable debido a su uso de drogas?	0	1
6. ¿Alguna vez su pareja (o familiares) se han quejado de su uso de drogas?	0	1
7. ¿Ha desatendido a su familia debido a su uso de drogas?	0	1
8. ¿Se ha implicado en actividades ilegales con el fin de obtener drogas?	0	1
9. ¿Alguna vez ha experimentado síntomas de abstinencia (sentirse enfermo) cuando dejo de usar drogas?	0	1
10. ¿Ha tenido problemas médicos como resultado de su uso de drogas (perdida de la memoria, hepatitis, convulsiones, hemorragia, etc.)?	0	1

**Score**	**Risk**	**Action**
0	Low Risk	Positive Reinforcement
1-2	At-Risk Use	Brief Intervention
3-5	Harmful Use	Referral to Brief Treatment
6+	Potential Dependent	Referral to Specialty Treatment

Registre la puntuación aquí

Date:___________ MRN______________________

Test de Identificación de Trastornos por consume de alcohol: version de entrevista

Lea las preguntas tal como están escritas. Registre las repuestas cuidadosamente. Empiece el AUDIT diciendo, “Ahora voy a hacerle algunas preguntas sobre su consume de bebidas alcohólicas durante el ultimo año.” Explique qué entiende por, “bebidas alcohólicas” utilizando ejemplos típicos como cerveza, vino, vodka, etc. Codifique las repuestas en terminus de consumiciones (“bebidas estándar”). Marque la cifra de la repuesta adecuada en el recuadro de la derecha.

6. ¿Con qué frecuencia consume alguna bebida alcoholic?<br>(5) Nunca [Pase a las preguntas 9-10]<br>(6) Una o menos veces al mes<br>(7) De 2 a 4 veces al mes<br>(8) De 2 a 3 veces a la semana<br>(9) 4 o más veces a la semana	6. ¿Con qué frequencia en el curso del último año ha necesitado beber en ayunas para recuperarse después de haber bebido mucho el día anterior?<br>(5) Nunca<br>(6) Menos de una vez al mes<br>(7) Mensualmente<br>(8) Semanalmente<br>(9) A diario o casi a diario
7. ¿Cuantas consumiciones de bebidas alcohólicas suele realizer en un día de consume normal?<br>(5) 1 or 2<br>(6) 3 or 4<br>(7) 5 or 6<br>(8) 7, 8, o 9<br>(9) 10 o más	7. ¿Con qué frecuencia en el curso del último año ha tenido remordimientos o sentimientos de culpa despúes de haber bebido?<br>(0) Nunca<br>(1) Menos de una vez al mes<br>(2) Mensualmente<br>(3) Semanalmente<br>(4) A diario o casi a diario
8. ¿Con qué frecuencia toma 6 o más bebidas alcohólicas en un solo día?<br>(0) Nunca<br>(1) Menos de una vez al mes<br>(2) Mensualmente<br>(3) Semanalmente<br>(4) A diario o casi a diario	8. ¿Con qué frequencia en el curso del último año no ha podido recorder lo que sucedió la noche anterior porque había estado bebiendo?<br>(0) Nunca<br>(1) Menos de una vez al mes<br>(2) Mensualmente<br>(3) Semanalmente<br>(4) A diario o casi a diario
9. ¿Con qué frecuencia en el curso de último año ha sido incapaz de parar de beber una vez había empezado?<br>(0) Nunca<br>(1) Menos de una vez al mes<br>(2) Mensualmente<br>(3) Semanalmente<br>(4) A diario o casi a diario	9. ¿Usted o alguna otra persona ha resultado herido porque usted había bebido?<br>(0) No<br>(2) Sí, pero no en el curso del último año<br>(4) Sí, el último año
10. ¿Con qué frecuencia en el curso del último año no pudo hacer lo que se esperaba de usted porque habia bebido?<br>(0) Nunca<br>(1) Menos de una vez al mes<br>(2) Mensualmente<br>(3) Semanalmente<br>(4) A diario o casi a diario	10. ¿Algún familiar, amigo, médico o professional sanitario ha mostrado preocupación por su consumo de bebidas alcohólicas o le han sugerido que deje de beber?<br>(0) No<br>(2) Sí, pero no en el curso del último año<br>(4) Sí, el último año

	AUDIT	DAST
Neg	0-7	0
BI	8-15	1-2
EBI	16-19	3-5
RT	20+	6+

Registre la puntuación aquí
