# Supplementary material for: Usability Testing of a National Substance Use Screening Tool Embedded in Electronic Health Records
Source: JMIR Hum Factors. 2016 Jul 8;3(2):e18. doi: 10.2196/humanfactors.5820 (PMC4958139; doi:10.2196/humanfactors.5820)
Supplement: Multimedia Appendix 2 [file humanfactors_v3i2e18_app2.pdf]

SCREENING

Patient SBIRT PreScreened and responses entered into SBIRT eCalcs Tool

Click **'CALCULATE'**
PreScreen score calculated
AUDIT-C*
DAST-1*
Tobacco

Click **'ADD to CHART'**
SBIRT PreScreen saved and result automatically entered into patient's chart

TASK + REASSIGNING

Task auto-generated for PreScreen POSITIVE and sent to **SCREENER's** Task List

Highlight individual Task and click **'REASSIGN'**

1. Assign to: **'TEAM'**
2. Select respective **'SBIRT Health Coach Team'**
   - 865, ACU, Glen Cove = 2001)
3. Priority: **'URGENT'** for any **AUDIT/DAST Positive***
   - SMOKING ONLY: leave priority as ASAP (default)
4. Feel free to add relevant comments in box (eg. Room#)
5. Click **'OK'**

SBIRT Health Coach views task in **'Team List'** and completes task
